# DeepEthoProfile—Rapid Behavior Recognition in Long-Term Recorded Home-Cage Mice

**DOI:** 10.1523/ENEURO.0369-24.2025

**Published:** 2025-07-08

**Authors:** Andrei Istudor, Alexej Schatz, York Winter

**Affiliations:** Humboldt-Universität zu Berlin, Berlin 10099, Germany

## Abstract

Animal behavior is crucial for understanding both normal brain function and dysfunction. To facilitate behavior analysis of mice within their home environments, we developed DeepEthoProfile, an open-source software powered by a deep convolutional neural network for efficient behavior classification. DeepEthoProfile requires no spatial cues for either training or processing and is designed to perform reliably under real laboratory conditions, tolerating variations in lighting and cage bedding. For data collection, we introduce EthoProfiler, a mobile cage rack system capable of simultaneously recording up to 10 singly housed mice. We used 36 h of manually annotated video data sampled in 5 min clips from a 48 h video database of 10 mice. This published dataset provides a reference that can facilitate further research. DeepEthoProfile achieved an overall classification accuracy of over 83%, comparable with human-level accuracy. The model also performed on par with other state-of-the-art solutions on another published dataset ( Jhuang et al., 2010). Designed for long-term experiments, DeepEthoProfile is highly efficient—capable of annotating nearly 2,000 frames per second and can be customized for various research needs.

## Significance Statement

DeepEthoProfile addresses a long-standing need for robust, automated analysis of rodent activity within undisturbed home cages. Powered by a deep convolutional neural network, this open-source system achieves near-human-level accuracy—over 83% on a newly published 36 h annotated dataset—and compares favorably with other state-of-the-art solutions on an established benchmark. Its tolerance to varying lighting, bedding arrangements, and mouse morphology makes it particularly suited to real-world laboratory conditions. By annotating nearly 2,000 frames per second, DeepEthoProfile accelerates high-throughput behavioral phenotyping and enables long-term investigations of day/night cycles, strain differences, and aging-related frailty. The use of a Docker-based pipeline eases adoption and maintenance with a minimum of requirements.

## Introduction

Mice and rats are central to biological and medical research. Long-term classification of behavioral activity is crucial for understanding mammalian brain function and investigating its dysfunction. In standard experimental setups, automated behavior evaluation is relatively straightforward when focusing on basic metrics such as general activity, body movement, and spatial usage (Contet et al., 2001; Tanaka et al., 2012; Lorbach et al., 2019; Ebbesen and Froemke, 2022). However, it becomes more challenging when it involves identifying specific behavioral actions in mice within their home cages, because mice lack distinct body features and can rapidly elongate or contract. Furthermore, standard animal welfare measures introduce complexities into home-cage-based video acquisition and data interpretation. The development of deep neural networks has been significantly advanced by improvements in consumer hardware, large databases, and deep learning libraries, leading to major progress in human action recognition (Karpathy et al., 2014; Tran et al., 2018). Although methods for human recognition are not directly transferable to rodent studies, they have opened new avenues in animal research. Techniques such as deep convolutional neural networks (CNNs), long short-term memory (LSTM) networks (Le and Murari, 2019), and optical flow features (Bohnslav et al., 2021) are now common for video annotating in both human and animal domains.

Behavior analysis requirements generally fall into two categories. On the one hand, researchers may need detailed kinematic data of body and limb movements of mice in specific experimental conditions. In this domain, DeepLabCut (Mathis et al., 2018) and SLEAP (Pereira et al., 2022) enable precise pose estimation across diverse settings. On the other hand, long-term observation in standard environments centers on daily behavioral sequences under unprovoked, natural conditions. The chaining of basic behaviors into a day-to-day sequence is influenced by genetics, environment, and disease. Identifying and understanding these behavioral elements and their timing offers insights into both healthy and disturbed patterns. In neurodegenerative disease research, such monitoring is critical for early symptom detection, often impossible by other methods, and for assessing treatment efficacy (Adamah-Biassi et al., 2013; Bains et al., 2018; Colomb and Winter, 2021). Moreover, automatic classification aligns with the 3R principles (replace, reduce, refine), potentially minimizing the number of animals used while improving data quality.

Quantifying daily routines in standard home cages presents unique obstacles. Mice frequently rearrange the bedding, altering the environment and complicating automatic detection. A commercial software suite (Adamah-Biassi et al., 2013) offers home-cage behavior analysis but is proprietary and expensive. Over a decade ago, an open-source solution from Tomaso Poggio’s lab at M.I.T. (Jhuang et al., 2010) was developed but is no longer maintained. The database from Jhuang et al. (2010) is still being used as a reference by various projects (Nwokedi et al., 2023; Jiang et al., 2017, 2019; Nguyen et al., 2019). Work from van Dam et al. (2020) relied on a multifiber network to classify overhead recordings of singly housed rats, while an approach integrating optical flow with a deep CNN showed promise for rodent and fly data (Bohnslav et al., 2021). For a more comprehensive review of recent approaches, see Segalin et al. (2021).

We developed a hardware and software solution, for extended recording and recognition of basic behaviors in singly housed mice in standard home cages. Our acquisition system, EthoProfiler, can record up to 10 cages at once. We used it to record 10 mice of two strains (different fur colors) for over 2 d each. Mice were individually housed with *ad libitum* access to food and water and a standard amount of bedding. We manually annotated single frames of 36 h of video from this data and then used those annotations to train and validate the DeepEthoProfile classification model. Our software excels in speed, accuracy, ease of use, and open-source availability, demonstrating strong performance on both our dataset and the previously published one (Jhuang et al., 2010).

## Material and Methods

We have developed an experimental system comprising a mobile, compact hardware platform, acquisition software, and processing software. DeepEthoProfile performs per-frame video annotations via a PyTorch-based deep convolutional neural network (CNN). The implementation processes short sequences of image frames as multichannel images. Aside from the input layer, this approach closely resembles standard image classification neural networks (Krizhevsky et al., 2017).

DeepEthoProfile runs in a Docker container environment, eliminating the need for special software installations. Processing is very fast and parallelizable. We ran six instances in parallel on a computer with an Intel i7-6700 processor and NVidia GTX 1080 graphics card (8 GB RAM), achieving over 1900 classified frames per second. Consequently, a 24 h video recorded at 25 fps can be annotated in under 20 min on 2016-era hardware.

### Acquisition system

**Figure 1.**
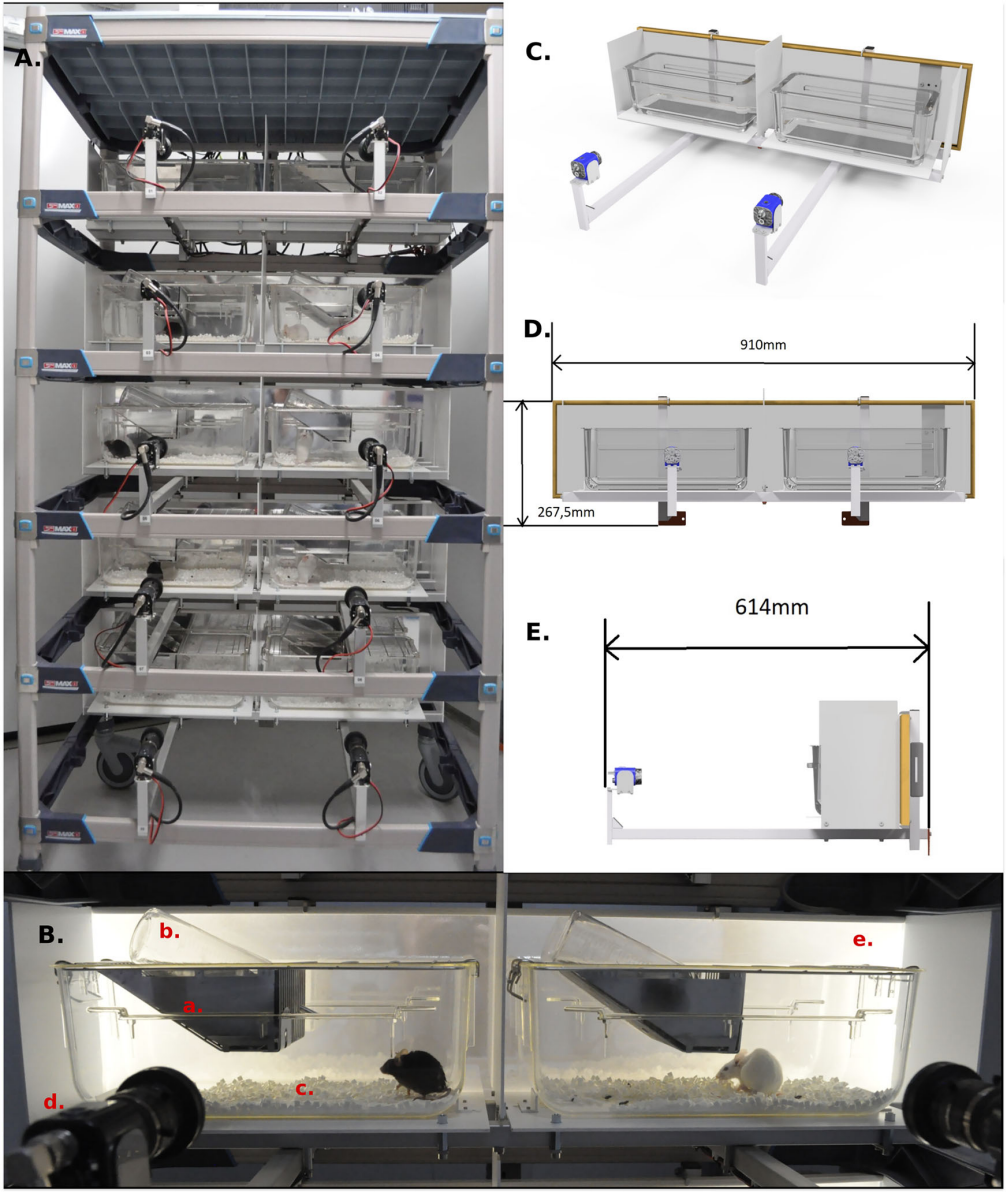
The EthoProfiler 10-cage data acquisition system. ***A***, Frontal view of the setup, showing video cameras at the front, two cages per shelf level, and a backlighting panel at the rear, all mounted on a MetroMax shelf. ***B***, Close-up of one shelf, with two adjacent Tecniplast 1145T cages housing mice of different strains (C57BL/6 on the left, SWISS on the right). Each cage contains the following: (a) a wire lid with *ad libitum* access to food, (b) a water bottle, and (c) paper bedding. Each camera (d) faces one cage, while the backlighting panel (e) provides illumination. Infrared background lighting in the processed videos was more uniform than in the daylight images shown here (Fig. 2). ***C–E***, 3D schematics of one shelf: (***C***) overview, (***D***) front view (similar to panel ***B***), and (***E***) side view.

For this study, we created a mobile and compact system to record long-term behavioral video of 10 singly housed mice. These 10 cages and their respective cameras are installed in a bottomless mobile rack (MetroMax i 5-Shelf), arranged in five rows of two cages each (Fig. 1*A*). We used conventional wire-lid home cages (Tecniplast Model 1145T, 369 × 165 × 132 mm, floor area 435 cm²). Their distinct long-and-narrow shape encourages left–right movement in front of the side-view camera (Fig. 1*B*). To differentiate feeding from drinking, we added a custom barrier in the food tray that confines pellet access to the left side, so pellet feeding is always on the left while drinking remains on the right.

Whereas some video methods advise minimal or no bedding for easier mouse detection, a practice that can cause stress and alter natural behavior, we used 200 ml of ALPHA-dri paper bedding (Tecnilab-BMI) and found our software to be robust despite bedding rearrangements. For illumination, we used backlighting panels across the camera’s field of view to reduce reflections. White plastic walls on each side of every cage prevent visual contact between neighboring cages and enhance uniform lighting. Though backlighting limits visible surface detail, it keeps the system compact. Direct front lighting would provide more detail but introduce reflections and complicate the design.

Given that mice are crepuscular or nocturnal and require a day/night cycle, our illumination panel contained two independent light sources of different wavelengths. Infrared light was always on for video acquisition while additional white light at ∼60 lux was switched on during the light phase (but remained invisible on video due to an IR filter). We used a Watec extreme low-light camera (WAT-902H2 Ultimate CCIR) at a small aperture for increased depth of field and with short exposure time to freeze motion, along with an infrared filter (Heliopan 5850) on the lens (Computar H0514MP2) that blocked visible light under 850 nm. The analog signals (25 fps@704 × 576) were digitized and simultaneously encoded using a dedicated Picolo U16 H.264 board (Euresys).

The recording output consists of MKV files, each up to six hours in length, containing an H.264-encoded video stream with a per frame timestamp. The acquisition software, written in C# and using DirectShow, is provided in our GitHub repository under “Capture.”

### Behavior database

**Figure 2.**
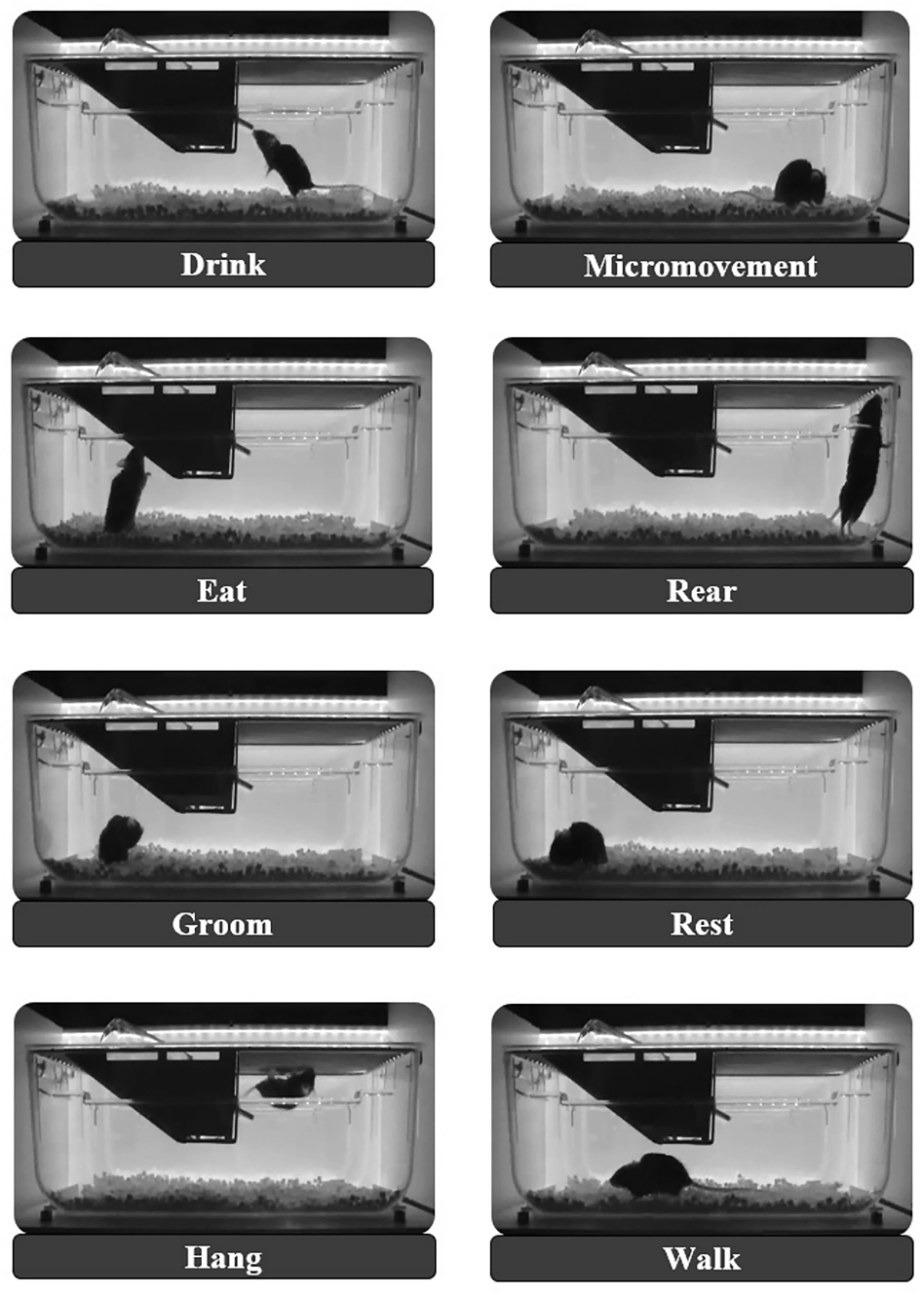
The eight basic behavior categories as defined in Jhuang et al. (2010). Representative frames from the annotated database are shown. Monochromatic images were captured using an infrared (IR) background light and a camera filter blocking wavelengths below 850 nm.

We studied 10 animals: five black C57BL/6NHsd (C57BL/6) females (Envigo) and five white RjOrl:SWISS (SWISS) females (Janvier), all 13 months old at recording. Data were collected for over 2 d. For manual annotation, each mouse had three or four 6 h recordings chosen to be evenly spaced across the 48 h period. In each selected video, 5 min intervals at the top of every half hour were annotated, yielding 60 annotated minutes per video and 180–240 min per mouse. We used the eight basic behavior categories described in Jhuang et al. (2010): eat, drink, groom, micromovement, rear, hang, walk, and rest (Fig. 2). Two trained biologists annotated half of the dataset each, labeling every frame with one behavior.

The dataset comprises 3,240,000 annotated frames, with their distribution shown in Figure 4. Because the mouse body has few distinct visual features under these illumination conditions, many frames appear highly similar. This limited distinctiveness stems from the mouse's anatomy, coat color, and the specific lighting setup. In addition, the mouse’s movement speed can vary considerably, and its overall size and shape can change drastically. During long-term recordings, behavior bouts may last from a few hundred milliseconds to several minutes, and mice frequently redistribute bedding on the cage floor. Despite these challenges, the large size and broad coverage of our database helped mitigate potential limitations. We anticipate that it will serve as a valuable reference for future methodological innovations.

To evaluate the consistency of the initial annotations, a third biologist reviewed two randomly selected sets of clips that had been annotated previously. Overall 35% of the original clips were reviewed, and in the following we refer to them as reviewed set 1 (20% of the original data) and reviewed set 2 (15% of the original data). Labels were changed where necessary, and the resulting confusion matrix (Fig. 5*C*) showed an overall agreement of labelled frames of 86.4% between the initial and the revised annotations and a macroaccuracy of 87.8%.

Additionally, in reviewed set 2 we introduced a new behavior category, “None,” for frames in which no behavior could be determined. These were primarily frames where the mouse was facing away from the camera but was not resting. This new category was applied to nearly 4% of frames in reviewed set 2.

Reviewed sets 1 and 2 covered one-third of the initial data and were added to our database for training and testing.

Annotations were done using a Python script, “AnnotationViewer,” included in our GitHub repository. It allows frame-by-frame playback, navigation, and annotation, saving results as CSV files.

### Processing software

DeepEthoProfiler is implemented in Python using PyTorch. Running the software requires Python 3, QT5, Docker, and CUDA. Processing occurs inside a Docker container, removing the need for specialized library installations. The application is compatible with modern Linux systems featuring NVIDIA GPUs that support CUDA 10.1 or higher.

The graphical user interface (GUI) is designed to be simple. Users can load videos one by one or add multiple files from a folder into a queue. A background process starts a separate Docker container for each file. If GPU memory allows, multiple containers run in parallel. The GUI shows a progress bar and completion percentage. All steps are automated requiring no extra user input. The output is a CSV with frame-by-frame annotations (including timestamps) that sit next to the processed video file.

### Overview

DeepEthoProfile uses a deep CNN to classify each video frame into a specific behavior. The design is inspired by standard image classification methods (Krizhevsky et al., 2017) and is similar to other 2D convolution approaches for video classification (Karpathy et al., 2014; Tran et al., 2018). The input is a stack of frames encoding temporal information in separate channels. A unique feature is the first convolution layer, which includes multiple asymmetrical filters. These filters act on the input, and their outputs are stacked for further processing.

### Input data format

Because recording illumination was monochromatic, we obtained single-channel visual images. The acquisition software converts these data to color before encoding and storing. During preprocessing each frame is reconverted to grayscale, resized to 256 × 256, and has its contrast enhanced. The pixel intensities are normalized to the [0,1] range. Eleven consecutive frames are then stacked into a single 11-channel image (stacked image) that will be the input for the CNN. This approach captures motion cues, offsetting the lack of distinct spatial features. The method from Jhuang et al. (2010) used nine frames equivalent to a 300 ms sequence at a recording rate of 30 frames per second (fps). We found that 11 frames gave a 4−5% accuracy gain, while 13 frames or more offered no further benefit or even reduced macroaccuracy. Thus, 11 frames (∼440 ms) was optimal, consistent with Wiltschko et al. (2015) . Different fps values may require adjusting the stack size.

### First layer

We applied batch normalization (BN) to the input before being forwarded to the four distinct rectangular kernels in the first layer of the CNN. These varying filters effectively capture both small and large movements and consider relative positions within the environment, removing the need for explicit cage or feeder location data. Each convolution is followed by a Rectified Linear Unit (ReLU), and the outputs are stacked in the same coordinate space.

### CNN description

Subsequent layers follow a standard pattern (Krizhevsky et al., 2017), with three additional convolution layers (each followed by a ReLU, and two fully connected layers). ReLU and BN boost training speed and help prevent overfitting.

**Figure 3.**
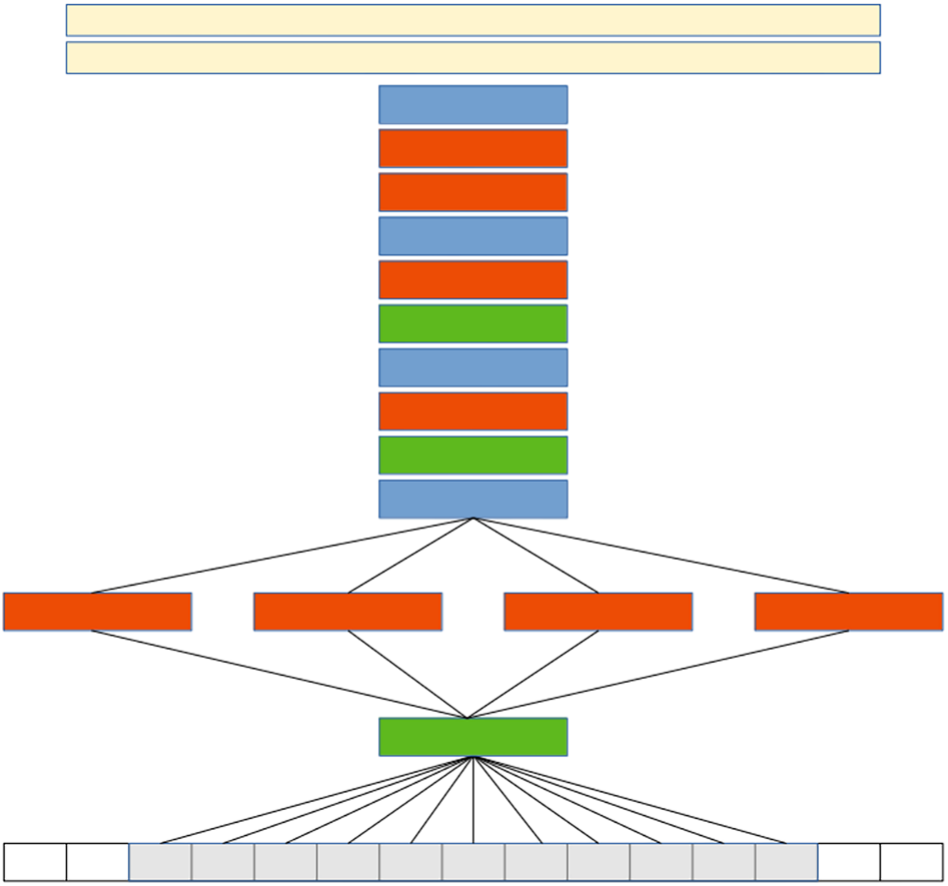
The DeepEthoProfile CNN model architecture. Convolutional layers are shown in red, normalization layers in green, pooling layers in blue, and fully connected layers in yellow. The gray boxes represent the 11 input frames.

The architecture of the CNN is summarized as follows *BN-(C(32, (11,1), 2), C(32, (1,11), 2), C(32, (7,3), 2), C(32, (3,7), 2))-P-BN-C(256, 5, 1)-P-BN-C(384, 3, 1)-P-C(512, 3, 1)-C(256, 3, 1)-P-FC(2048)-FC(2048)* (Fig. 3). The max-pooling layers (*P*) have a spacing of 2 and size of 3. *C(d, f, s)* denotes a convolutional layer with *d* filters of size *f* × *f*, applied to the input with stride *s*. *BN* represents a batch normalization layer*. FC(n)* represents a fully connected layer with n nodes. Each convolutional and *FC* layer was followed by a ReLU. A one-dimensional dropout of 0.5 was applied before each fully connected layer, reducing coadaptation of neurons (Hinton et al., 2012). There is a final *FC* layer with the number of nodes corresponding to the number of classified behaviors, in our case eight.

### Training

We trained the network on the 5 min clips, each frame labeled with one of the eight behaviors. For an 11-frame stack, the target label was the majority annotation within those frames. If a tie arose, the central frame’s label was used. This eliminated brief and potentially invalid behaviors, minimizing the impact of noise in manual annotations while preserving the accuracy of the trained model.

**Figure 4.**
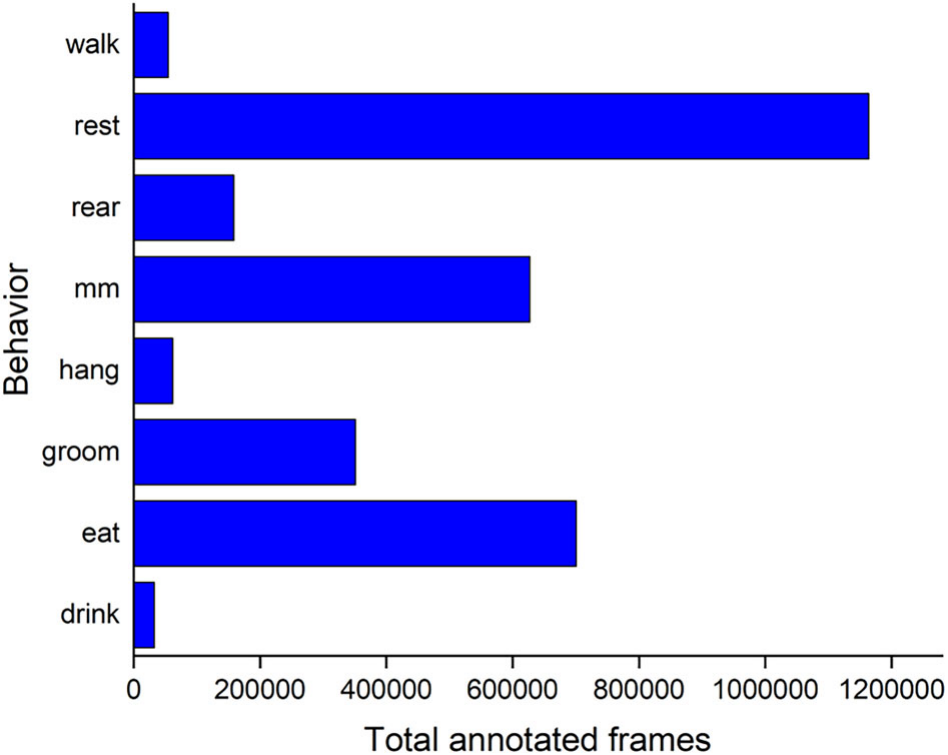
Distribution of annotated frames for each behavior category. The dataset comprises 3,240,000 frames, equivalent to 36 h of video material.

Because the dataset was imbalanced (Fig. 4), we implemented a dynamic balancing approach with random sampling that favored underrepresented behaviors. Every minibatch contained an example from each behavior and exactly one from the least prevalent behavior, in this case “drink.” The sampling probability was calculated to ensure that there were at least twice as many occurrences of every other behavior in each training epoch. We used a minibatch size of 16 and attempted to pick each of the stacked images from a different training clip. Some behavior bouts are long and repetitive. This approach tried to avoid having stacked images that were too similar in the same minibatch to avoid overfitting. Training used cross-entropy loss with stochastic gradient descent (SGD), an initial learning rate of 0.03, and exponential decay at 0.99. We also applied standard augmentations (random shifts and horizontal flips) to reduce overfitting.

When available, the data of the reviewed sets was used instead of the initial annotations. For the data from the reviewed set 2 with the “None” behavior, frames of that new category were excluded from the training process. The database does not contain enough samples of “None” to classify it. Thus, reviewed set 2 was not used for model validation.

### Processing

At inference, one classification per 11-frame stack was applied to all 11 frames. This enforced a minimum 440 ms behavior duration, reducing noise and improving speed. Though transitions may shift by up to five frames (∼200 ms), this is negligible in day-scale analyses.

### Code accessibility

The source code for DeepEthoProfile is accessible via the GitHub repository under the GPL-3.0 license at https://github.com/WinterLab-Berlin/DeepEthoProfile. The software runs on a PC installed with an Ubuntu 24.04 operating system, provided a Nvidia graphics card and the corresponding CUDA drivers are installed. Additional information and software requirements are found on the GitHub page. The code is available as Extended Data. The model used by the processing software will be downloaded by the starting script from https://doi.org/10.5281/zenodo.14827053.

The acquisition software runs on Windows 10 and can be found at https://github.com/WinterLab-Berlin/DeepEthoProfile/tree/main/Capture.

The database with all the annotations is published under the MIT license and can be found at https://doi.org/10.5281/zenodo.14782614.

10.1523/ENEURO.0369-24.2025.d1 Extended Data Download Extended Data, ZIP file.

## Results

### Validation

**Figure 5.**
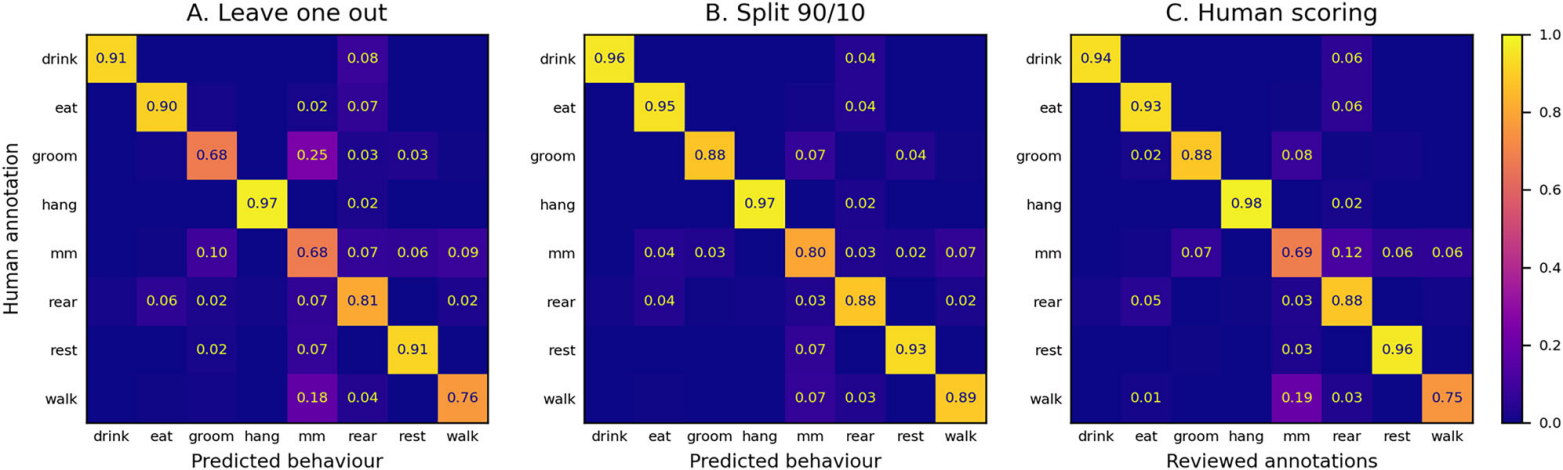
Confusion matrices for animal behavior detection rates using data acquired with EthoProfiler. Rows represent the manually annotated behaviors. ***A***, The sum of confusion matrices from 10 leave-one-out tests. In each test, the model was trained on data from nine mice and tested on the remaining mouse. ***B***, Results of training on 90% of the data and testing on the remaining 10%, with all reviewed annotations included in the training set. ***C***, Results from the partial review of the initial human annotation data, where columns represent the reviewed behavior annotations.

We performed leave-one-out cross-validation, training with the data from nine mice, and testing on the data from the one that was left out. Repeating this for all mice, we trained for 40 epochs in each case. Summing and normalizing results gave an overall frame-level accuracy of 83.6% and a macroaccuracy of 82.9% (Fig. 5*A*).

The difficulty in accurately classifying frames as micromovement largely stems from its definition as “small movements of the animal's head or limbs” (Jhuang et al., 2010), which is open to misinterpretation. This category exhibited low accuracy in both our human review of annotations (Fig. 5*C*) and the review performed in Jhuang et al. (2010) (Fig. 7*C*). Moreover, when we examined frames labeled as “None” during our database review, over 91% had originally been annotated as either groom or micromovement. More than 8% of the previously labeled micromovement frames and 9% of the grooming frames aligned with the “None” classification. These findings highlight a limitation of our side-view recordings, which was not fully appreciated during the initial annotation phase. With limited visual information, the annotator had to infer the animal’s behavior in ambiguous frames, often splitting the same cues between two categories and preventing the model from learning effectively.

Such ambiguous frames significantly contributed to the difficulty in distinguishing micromovement from grooming.

Misclassification between micromovement and walk arose especially when the mouse moved very slowly, for very short periods of time, or changed body length. Similar ambiguities appeared in the revised confusion matrix (Fig. 5*C*).

When comparing strains, the classification on C57BL/6 mice had lower accuracy than on SWISS mice, an outcome mirrored in the annotation review (Table 1).

**Table 1.** Comparison of the accuracy of the model across mouse strains with leave-one-out test results

Mouse Strain	DeepEthoProfile		Review agreement	
	Frame accuracy	Macroaccuracy	Frame accuracy	Macroaccuracy
C57BL/6	80%	81%	84.4%	87.0%
SWISS	85.5%	84.5%	87.8%	88.6%

Despite these issues, the cross-validation performance remained close to human annotation quality.

### DeepEthoProfile model

**Figure 6.**
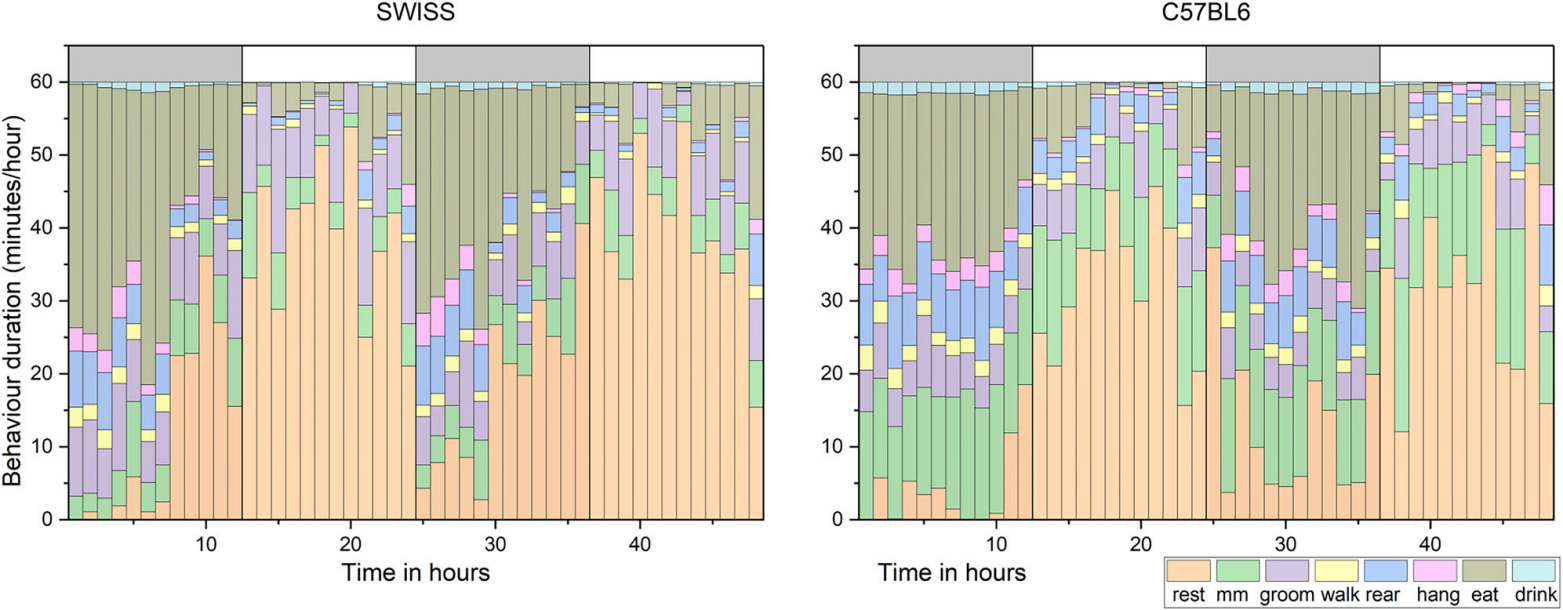
Mean behavior durations monitored over 48 h. ***A***, Data from SWISS mice (*n* = 5). ***B***, Data from C57BL/6 mice (*n* = 5). The data were acquired using the EthoProfiler setup and processed with DeepEthoProfiler. The shaded bar at the top of each figure indicates the dark phase. Data from the first hour after introducing the mice to their new cages were excluded.

We trained the final model on 90% of the clips and tested on the remaining 10%. All revised annotations were included in the training set for best results. The frames corresponding to the “None” category were ignored to avoid inconsistencies. The confusion matrix had a frame-level accuracy of 90.6% (Fig. 5*B*). This model is used by the DeepEthoProfile software. We used it to process the 2 d of video data from which our annotated database was previously selected. Results (Fig. 6) illustrate behavioral differences between strains and their adaptation to the day/night cycle.

### Comparison

To assess efficiency, we tested the model on the database published in Jhuang et al. (2010), a well-established benchmark used by several state-of-the-art methods (Nwokedi et al., 2023; Jiang et al., 2017, 2019; Nguyen et al., 2019). This dataset has two annotation sets: a “full database” of 12 videos (every frame labeled) and a “clipped database” of short, unambiguous segments.

**Figure 7.**
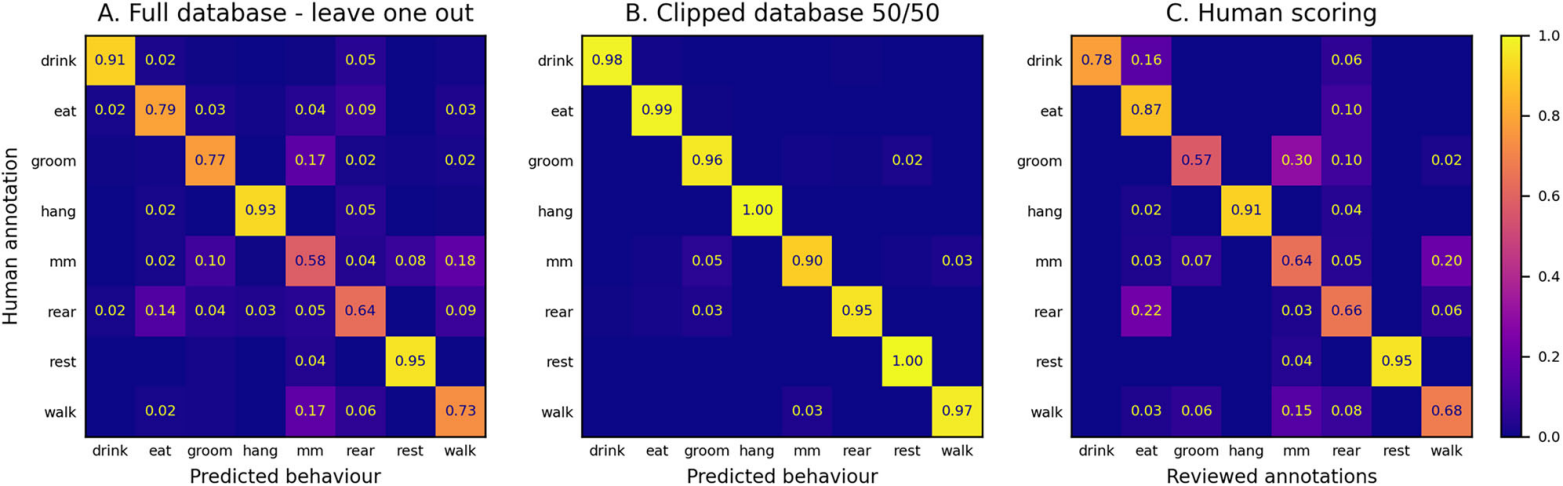
Confusion matrices for animal behavior detection rates using the dataset from Jhuang et al. (2010). Rows represent the manually annotated behaviors. ***A***, The sum of confusion matrices from 12 leave-one-out tests on the “full database.” In each test, the model was trained on data from 11 videos and tested on the remaining video. ***B***, The sum of confusion matrices from five tests on the “clipped database.” In each test, the model was trained on 50% of the videos (randomly selected) and tested on the remaining 50%. ***C***, Results from the partial review of the initial human annotation, as originally published in Jhuang et al. (2010).

We followed the same protocols used in prior studies. On the full database, we applied leave-one-out (training on 11 videos, testing on the remaining one) for 50 epochs each, obtaining 74.2% accuracy over frames and 78.7% macroaccuracy (Fig. 7*A*). A separate human annotation review in Jhuang et al. (2010) yielded 73% (frames) and 79% (macro) agreement, indicating that our results were close to human-level performance. On the clipped database, we split data 50/50 for training and testing, repeating this process five times (30 epochs each). Combined, we reached 97.6% overall accuracy and 96.8% macroaccuracy (Fig. 7*B*). Table 2 compares our results to other published methods.

**Table 2.** Representation of previously published classification models tested on the data from Jhuang et al. (2010) alongside results obtained with DeepEthoProfile

Method	Frame Acc (%)	Macro Acc (%)	Accuracy for each behavior (%)							
			Drink	Eat	Groom	Hang	Mm	Rear	Rest	Walk
Full database										
DeepEthoProfile	74.2	78.7	91	90	70	97	66	80	91	77
Jhuang et al. (2010)	78.3	76.4	72	75	70	92	83	70	94	55
Human (3)	71.6	75.8	78	87	57	91	64	66	95	68
Jiang et al. (2017)	72.9									
Jiang et al. (2019)	81.5	79.2	60.3	81.1	82.2	95.8	74.6	74.9	97.1	67.7
CleverSys (3)	60.9	64.0	63	73	30	82	64	35	96	69
Le and Murari (2019)	73.5	76.5								
Clipped database										
DeepEthoProfile	97.6	96.8	98	99	96	100	90	95	100	97
Jhuang et al. (2010)	93									
Jiang et al. (2017)	95.9	90.7	72.4	95.7	97.4	97.6	69.8	94.9	99.5	98.3
Dollar (2)	82.2	70.5	41	69	88.4	80.8	32.2	57.9	98.8	96.1
Wang et al. (2015) (1)	96.1	88.5	45.9	92	98.3	98.8	80.6	95.2	99	97.9
Wang et al. (2017) (1)	94.5	87.6	50.8	90.1	97	96	77.8	93.2	97.1	99.1
Jiang et al. (2019)	97.9	94.3	77	97.3	99.1	99.2	86.1	96.7	99.1	99.6
I3D (4)	96.9									
R(2 + 1)D (4)	96.3									
Nwokedi et al. (2023) (5)	82									

Results marked with (1) were published in Jiang et al. (2019), those with (2) were published in Jiang et al. (2017), and those with (3) appeared in Jhuang et al. (2010). The methods marked with (4) were tested in Nguyen et al. (2019). The results from (5) were trained on the “full database” and tested on the “clipped database”.

DeepEthoProfile performs on par with or better than most current techniques, especially on drink, though it struggles with micromovement in the full database, where annotations are often inconsistent. Notably, the agreement on micromovement behavior between human reviewers was just 64% in Jhuang et al. (2010).

## Discussion

We present a new open-source method to classify single-housed mouse behavior in standard home cages over extended periods. Our system is fast, robust to bedding changes, and well suited for long-term data collection. We also provide the dataset used to train and validate the model. To our knowledge, this dataset is the first freely available long-term video recording with frame-by-frame mouse behavior annotations. Our classification method was validated on the data from Jhuang et al. (2010), and it performed comparably with other leading systems. However, those videos were recorded in conditions that differ from standard husbandry: there is minimal bedding, uniform top illumination, and they are relatively short. Often the animals are in exploratory mode typical of a newly introduced cage. We have explicitly ignored the first hours of our more than 2 d recording to avoid that behavior.

Automated observation of animals in their usual habitat supports 3R principles by reducing negative impact on animals or refinement of animal welfare, and enhancing research efficiency. This noninvasive approach captures normal behavior spectra without causing stress or disturbance, potentially lowering the number of animals needed for invasive procedure. DeepEthoProfile can be adapted to be used on existing video databases to extract additional data with a high degree of consistency.

In typical biology laboratory settings, users found our system intuitive and easy to use, with classification performance comparable to human annotators. We look forward to further develop DeepEthoProfile to meet additional requirements and to adapt it to diverse experimental environments.

## Synthesis

Reviewing Editor: Nathalie Ginovart, University of Geneva Decisions are customarily a result of the Reviewing Editor and the peer reviewers coming together and discussing their recommendations until a consensus is reached. When revisions are invited, a fact-based synthesis statement explaining their decision and outlining what is needed to prepare a revision will be listed below. The following reviewer(s) agreed to reveal their identity: NONE.

The authors have constructively revised their manuscript, and this revised version is significantly improved over the initial submission. However, the first reviewer highlighted a few minor issues that need to be addressed before final acceptance.

Moreover, before final acceptance, authors must also comply with eNeuro's policy regarding studies using custom code. The code must be available upon acceptance and publication of the manuscript. As per eNeuro's Instructions to authors, code files should be deposited in a suitable repository such as GitHub, ModelDB, BioModels, CellML, or Visiome. The "Code Accessibility" section should therefore indicate how the code can be accessed and provide a repository link, including any accession numbers or restrictions, as well as the type of computer and operating system used to run the code.

Please find the reviewer's comments below:

Reviewer 1:

The manuscript is a marked improvement on the original, with a much better structure and more methodological details. A comparison with previous methods on the Jhuang et al. dataset is provided as well. I have some minor comments:

- Lines 181 - 188 need proper mathematical formatting, and the architecture should probably be provided as a table and/or diagram.

- Typo on Line 120 "aolder" -> "an older"?
