# Supplementary figures and images for: DeepEthoProfile—Rapid Behavior Recognition in Long-Term Recorded Home-Cage Mice
Source: eNeuro. 2025 Jul 8;12(7):ENEURO.0369-24.2025. doi: 10.1523/ENEURO.0369-24.2025 (PMC12265860; doi:10.1523/ENEURO.0369-24.2025)

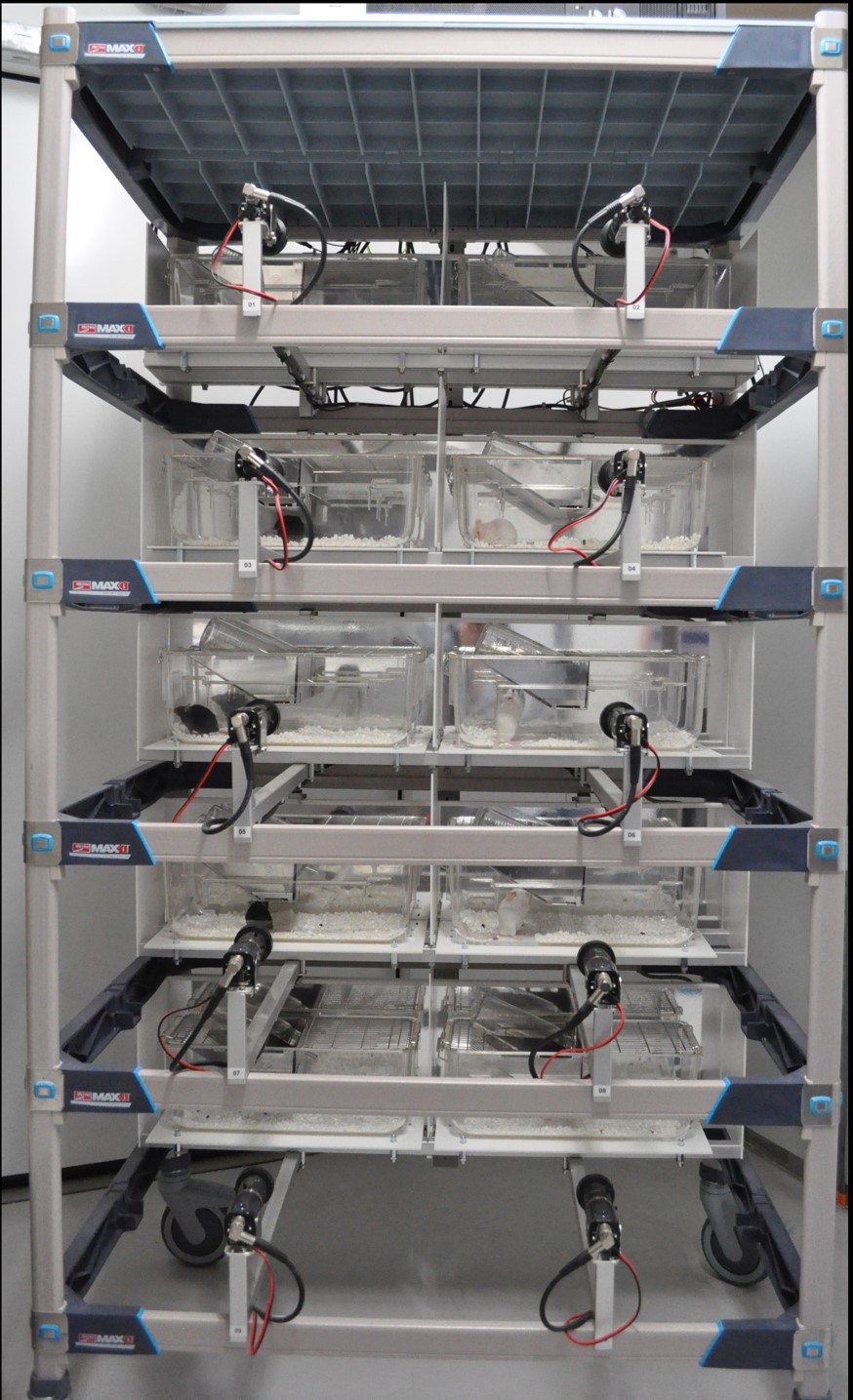

Supplement: Extended Data — Download Extended Data, ZIP file. [file eneuro-12-ENEURO.0369-24.2025-s001.zip › DeepEthoProfile-main/pics/10x.jpg]

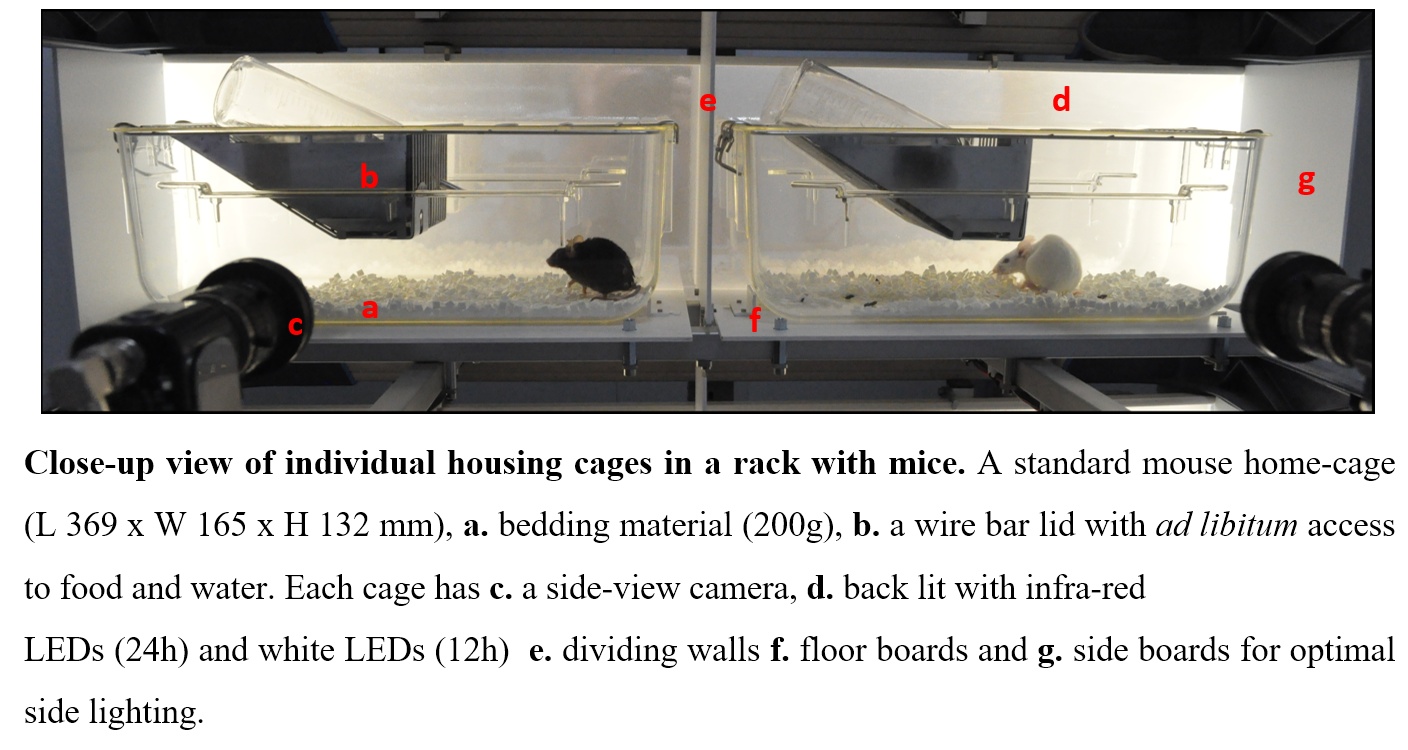

Supplement: Extended Data — Download Extended Data, ZIP file. [file eneuro-12-ENEURO.0369-24.2025-s001.zip › DeepEthoProfile-main/pics/10x_Detail_notes.jpg]

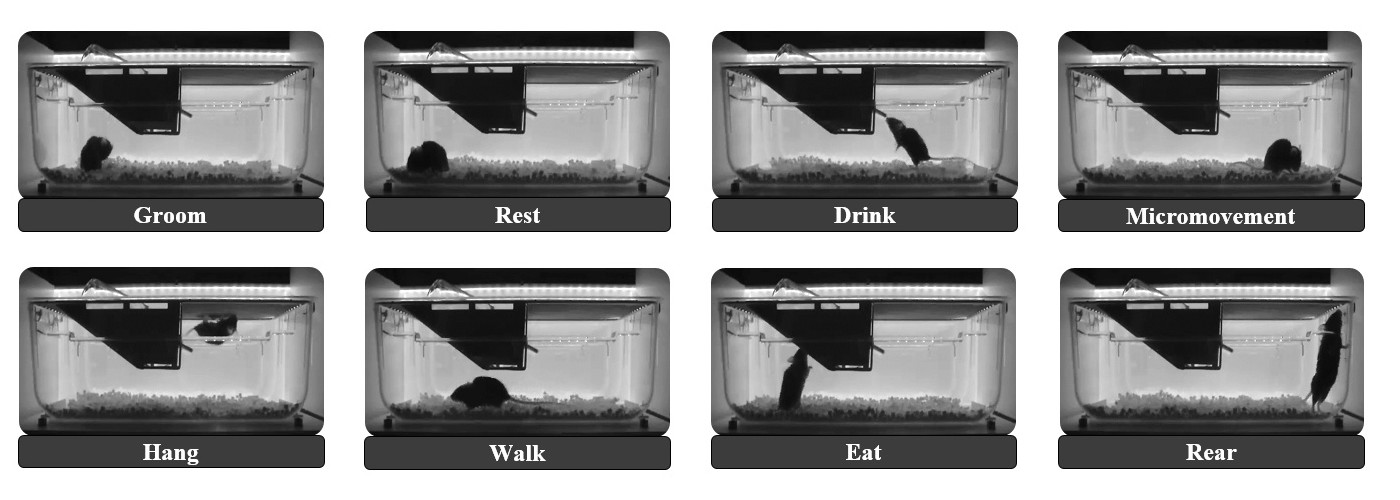

Supplement: Extended Data — Download Extended Data, ZIP file. [file eneuro-12-ENEURO.0369-24.2025-s001.zip › DeepEthoProfile-main/pics/behaviours_H.jpg]
